# Thermally Activated Delayed Fluorescence Host for High Performance Organic Light-Emitting Diodes

**DOI:** 10.1038/s41598-018-27238-y

**Published:** 2018-06-11

**Authors:** Lu Zhang, Kok Wai Cheah

**Affiliations:** Department of Physics and Institute of Advanced Materials, Hong Kong Baptist University, Kowloon Tong, Hong Kong SAR China

## Abstract

Thermally activated delayed florescence (TADF) materials can be an efficient host in organic LED (OLED). It is because it is possible to couple energetically the emission energy level of a dopant to the energy levels in the TADF material. In this work fluorescent emitters 2,3,6,7-tetrahydro-1,1,7,7,-tetramethyl-1H,5 H,11H-10-(2-benzothiazolyl)quinolizino-9,9a,1gh coumarin (c545t) and 5,6,11,12-tetraphenyltetracene (rubrene) were used as dopants in a blended TADF host; tris(4-carbazoyl-9-ylphenyl)amine (TCTA) with 2,4,6-tris(3′-(pyridin-3-yl)biphenyl-3-yl)-1,3,5-triazine (Tm3PyBPZ). The blended TADF host has an energy difference between the singlet and triplet excited states (ΔE_ST_) around 27 meV with the yield of reverse intersystem crossing (Ф_RISC_) nearly 100%. This high Ф_RISC_ yield enhances the OLED performance with the c545t doped OLED having 11.9% external quantum efficiency and 10% for the rubrene doped OLED.

## Introduction

When an organic light-emitting diode (OLED) is working under the electric field, the holes and electrons are injected from the electrodes and then form singlet excitons and triplet excitons in the emissive layer (EML) with the ratio of approximately 25%:75% from spin statistics. In a conventional fluorescent OLED, the 75% triplet excitons are generally non-emissive at room temperature, which limits the internal quantum efficiency (IQE) to be only 25%. By introducing heavy-metal induced spin-orbit coupling, the transitions between singlet and triplet states are possible which makes it possible to utilize both singlet and triplet excitons^1^. However, these transition metals cause other concerns such as high cost and environmental pollution. In recent years, a new approach to efficiently utilize the triplet excitons generated in the OLED has drawn a lot of attention, which is thermally activated delayed fluorescence (TADF); metal-free TADF fluorescent materials can harvest both singlet and triplet excitons, which greatly improves the IQE compared to the conventional fluorescent materials^2^. For a TADF material, the energy difference between singlet excited state and triplet excited state (ΔE_ST_) is usually very small (<0.1 eV). Therefore, the triplet excitons can be converted to singlet excitons via reverse intersystem crossing (RISC) with the assistance of thermal energy, which makes it possible to realize 100% IQE without using heavy metals^3^.

A small ΔE_ST_ is required for a TADF material, which enables a high RISC rate constant (k_RISC_) according to the equation as follows:

1 $${k}_{RISC}=Aexp(-\frac{{\rm{\Delta }}{E}_{ST}}{{k}_{B}T})$$

where A is a constant, k_B_ is the Boltzmann constant, and T is the temperature^4^. Therefore, the RISC can efficiently compete with other non-radiative decay such as internal conversion (IC). Since ΔE_ST_ scales with the spatial overlap of the highest occupied molecular orbital (HOMO) and lowest unoccupied molecular orbital (LUMO) wave functions Ψ_HOMO_ and Ψ_LUMO_ as shown in equation (2), the HOMO and LUMO of TADF materials are designed to be separately located on donor moiety and acceptor moiety, respectively, which results in a small ΔE_ST_ while maintaining a high fluorescent quantum yield^5,6^.

2 $${\rm{\Delta }}{E}_{ST}\propto {\int }^{}{\Psi }_{HOMO}(r){\Psi }_{LUMO}(r){d}_{r}^{3}$$

A small ΔE_ST_ can also be realized in some intermolecular excited-state complex (exciplex) formed between electron-donating molecule and electron-accepting molecule^7,8^. Therefore, they are good candidates for triplet harvesting through RISC. In an exciplex exhibiting TADF, the HOMO and LUMO are mainly located on donor and acceptor molecules, respectively. This kind of TADF material can be functioned as the emitter or as the host for some conventional fluorescent emitter to improve the IQE^9,10^.

## Results

In our earlier work, we have demonstrated efficient TADF OLEDs based on the exciplex forming between electron-donating material tris(4-carbazoyl-9-ylphenyl)amine (TCTA) and electron-accepting material 2,4,6-tris(3′-(pyridin-3-yl)biphenyl-3-yl)-1,3,5-triazine (Tm3PyBPZ)^11^. The exciplex shows a green emission with the peak wavelength around 514 nm. The maximum efficiencies of OLEDs based on TCTA:Tm3PyBPZ (1:1) are 13.1% and 53.4 lm/W under low turn-on voltage of only 2.4 V.

Here we estimate the ΔE_ST_ of TCTA:Tm3PyBPZ; time-resolved photoluminescence (PL) characteristics of TCTA:Tm3PyBPZ (1:1, 50 nm) film from 205 K to 295 K were measured by exciting the sample with a pulsed Nd:YAG laser. Since the emission of TADF material consists of prompt fluorescence (PF) that happened immediately after the excitation and delayed fluorescence (DF) through the RISC, the time-resolved PL characteristics were fitted by bi-exponential decay as shown in equation (3):

3 $$I={A}_{p}{e}^{-\frac{t}{{\tau }_{p}}}+{A}_{d}{e}^{-\frac{t}{{\tau }_{d}}}$$

where A_p_ and A_d_ are the proportional quantities of PF and DF, respectively. τ_p_ and τ_d_ are the lifetimes of PF and DF, respectively. The ratio between DF and PF (Ф_d_/Ф_p_) at different temperatures can be determined by the integral of the DF and PF components according to equation (4). The fitting results and the calculated Ф_d_/Ф_p_ are shown in Table 1.

4 $$\frac{{{\rm{\Phi }}}_{d}}{{{\rm{\Phi }}}_{p}}=\frac{{A}_{d}{\tau }_{d}}{{A}_{p}{\tau }_{p}}$$

The fluorescence yield of TADF emitters (Ф_F_) is described by equation (5), which includes recycling of singlet and triplet states.

5 $${{\rm{\Phi }}}_{F}={{\rm{\Phi }}}_{p}+{{\rm{\Phi }}}_{d}=\sum _{i=0}^{n}{{\rm{\Phi }}}_{p}{({{\rm{\Phi }}}_{ISC}{{\rm{\Phi }}}_{RISC})}^{i}={{\rm{\Phi }}}_{p}\frac{1}{1-{{\rm{\Phi }}}_{ISC}{{\rm{\Phi }}}_{RISC}}$$

Therefore, Фd/Фp can be expressed in terms of Φ_ISC_ and Φ_RISC_ as shown in equation (6).

6 $$\frac{{{\rm{\Phi }}}_{d}}{{{\rm{\Phi }}}_{p}}=\frac{1}{1-{{\rm{\Phi }}}_{ISC}{{\rm{\Phi }}}_{RISC}}-1$$

For Фd/Фp ≈ 4, Ф_ISC_Ф_RISC_ is around 0.8. Since Ф_ISC_^max^ = 1 − Фp, therefore Ф_RISC_ is close to 1^12,13^. In that case, the rate of RISC (k_RISC_) can be simply expressed by equation (7):

7 $${k}_{RISC}=\frac{1}{{\tau }_{d}}(\frac{{{\rm{\Phi }}}_{p}+{{\rm{\Phi }}}_{d}}{{{\rm{\Phi }}}_{p}})$$

**Figure 1:**
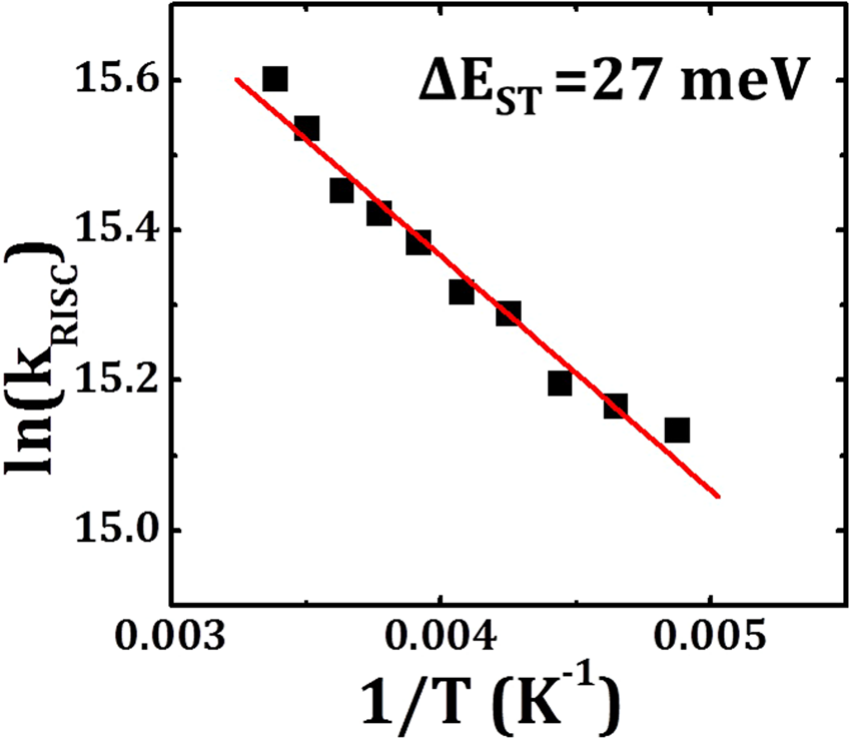
Arrhenius plot of the RISC rate constant of TCTA:Tm3PyBPZ (1:1, 50 nm) film. The square root of mean square error is 0.029.

Thus, k_RISC_ can be derived from Ф_d_/Ф_p_ and τ_d_.^[12]^ In this work, Ф_d_/Ф_p_ was obtained through equations (3) and (4) and is above 6 as shown in Table 1; thus the k_RISC_ can be calculated from equation (7) and shown in Table 1. When the temperature was decreased from 295 K to 205 K, k_RISC_ decreased from 6.0 × 10^6^ s^−1^ to 3.7 × 10^6^ s^−1^. Since k_RISC_ is related to ΔE_ST_ and temperature (equation 1), the activation energy can be estimated to be 27 meV from the Arrhenius plot as shown in Fig. 1.

**Table 1:** The time-resolved PL characteristics, the ratio between DF and PF (Ф_d_/Ф_p_) and the RISC rate constant (k_RISC_) of a TCTA:Tm3PyBPZ (1:1, 50 nm) film at different temperatures.

Temperature (K)	A_p_	τ_p_ (ns)	A_d_	τ_d_ (μs)	Ф_d_/Ф_p_	k_RISC_ (s^−1^)
205	0.1467	20.7	0.0100	2.3	7.68	3.7 × 10^6^
215	0.1599	18.0	0.0098	2.2	7.41	3.9 × 10^6^
225	0.1506	18.5	0.0097	2.1	7.36	4.0 × 10^6^
235	0.1202	20.7	0.0096	2.0	7.69	4.4 × 10^6^
245	0.1113	19.7	0.0086	1.8	6.96	4.5 × 10^6^
255	0.0975	20.8	0.0085	1.7	6.97	4.8 × 10^6^
265	0.0847	22.0	0.0081	1.6	6.89	5.0 × 10^6^
275	0.0798	21.7	0.0077	1.4	6.35	5.1 × 10^6^
285	0.0582	22.2	0.0062	1.3	6.22	5.6 × 10^6^
295	0.0428	24.7	0.0054	1.2	6.40	6.0 × 10^6^

The TADF material can also function as host for conventional fluorescent emitter improving the IQE. As shown in Fig. S1, the triplet excitons formed at the exciplex host can be converted to singlet excitons by RISC. Then the additional energy is transferred from singlet energy level of the host to the singlet energy level of the fluorescent emitter to increase the singlet excitons population formed on emitter.

**Figure 2:**
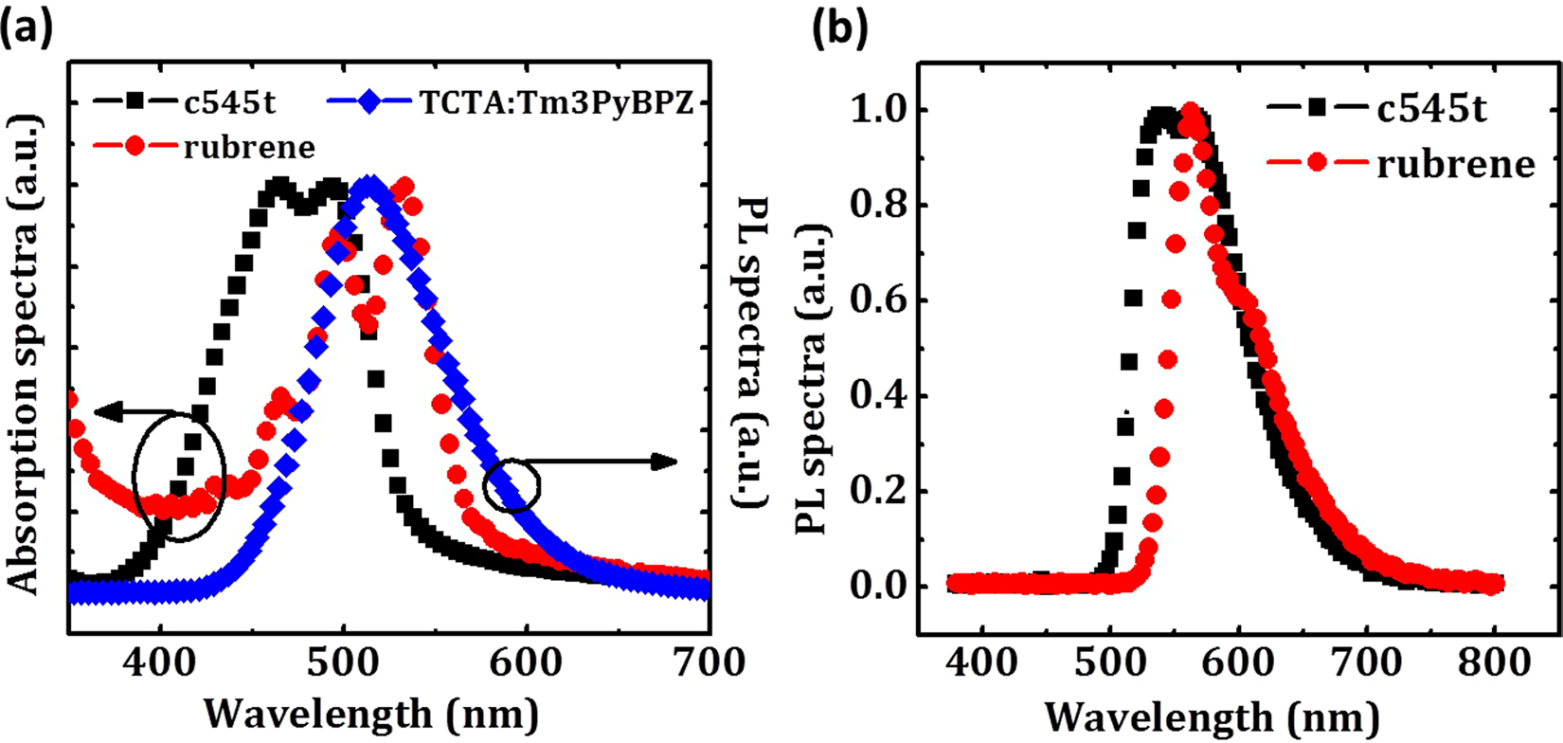
(**a**) absorption spectra of c545t and rubrene and emission spectrum of exciplex TADF host based on TCTA:Tm3PyBPZ (1:1) in solid state. (**b**) PL spectra of c545t and rubrene in solid state.

OLEDs were fabricated using TCTA:Tm3PyBPZ (1:1) exciplex as host for 2,3,6,7-tetrahydro-1,1,7,7,-tetramethyl-1H,5H,11H-10-(2-benzothiazolyl) quinolizino-9,9a,1gh coumarin (c545t) and 5,6,11,12-tetraphenyltetracene (rubrene). The chemical structures of TCTA, Tm3PyBPZ, c545t and rubrene are shown in Fig. S2. The energy level diagrams are shown in Fig. S3. The HOMO and LUMO levels of c545t and rubrene are near to those of the exciplex which minimize the exciton trapping and facilitate the transfer of excitation energy^14–16^. The absorption spectra of the dopants are well overlapped with the emission of the exciplex host as shown in Fig. 2a, which enables efficient Förster energy transfer from the host to the dopants. Figure 2b shows the PL spectra of thin films of c545t (50 nm) and rubrene (50 nm). The peak wavelengths of c545t and rubrene are 564 nm and 563 nm, respectively.

The OLED devices with a structure of 1,4,5,8,9,11-hexaazatriphenylenehexacarbonitrile [Hat(CN)_6_, 5 nm]/1,1-bis(4-(N,N-di(p-tolyl)-amino)phenyl)cyclohexane [TAPC, 65 nm]/EML [10 nm]/Tm3PyBPZ [50 nm]/8-hydroxy-quinolinato lithium [Liq, 2 nm]/Al [100 nm] were fabricated onto indium tin oxide (ITO) glass substrate (sheet resistance ~ 18 Ω/square). For the EML, the host was exciplex forming by co-evaporating TCTA and Tm3PyBPZ with the ratio of 1:1. Four OLEDs were fabricated: for Device A and B, the EML were c545t doped with host and the concentrations are 0.5% and 1%, respectively; for Device C and D, the EML were rubrene doped with host and the concentrations are 0.5% and 1%, respectively. Low doping concentrations were used in the EML to reduce the triplet-triplet energy transfer from the host to the dopants.

**Figure 3:**
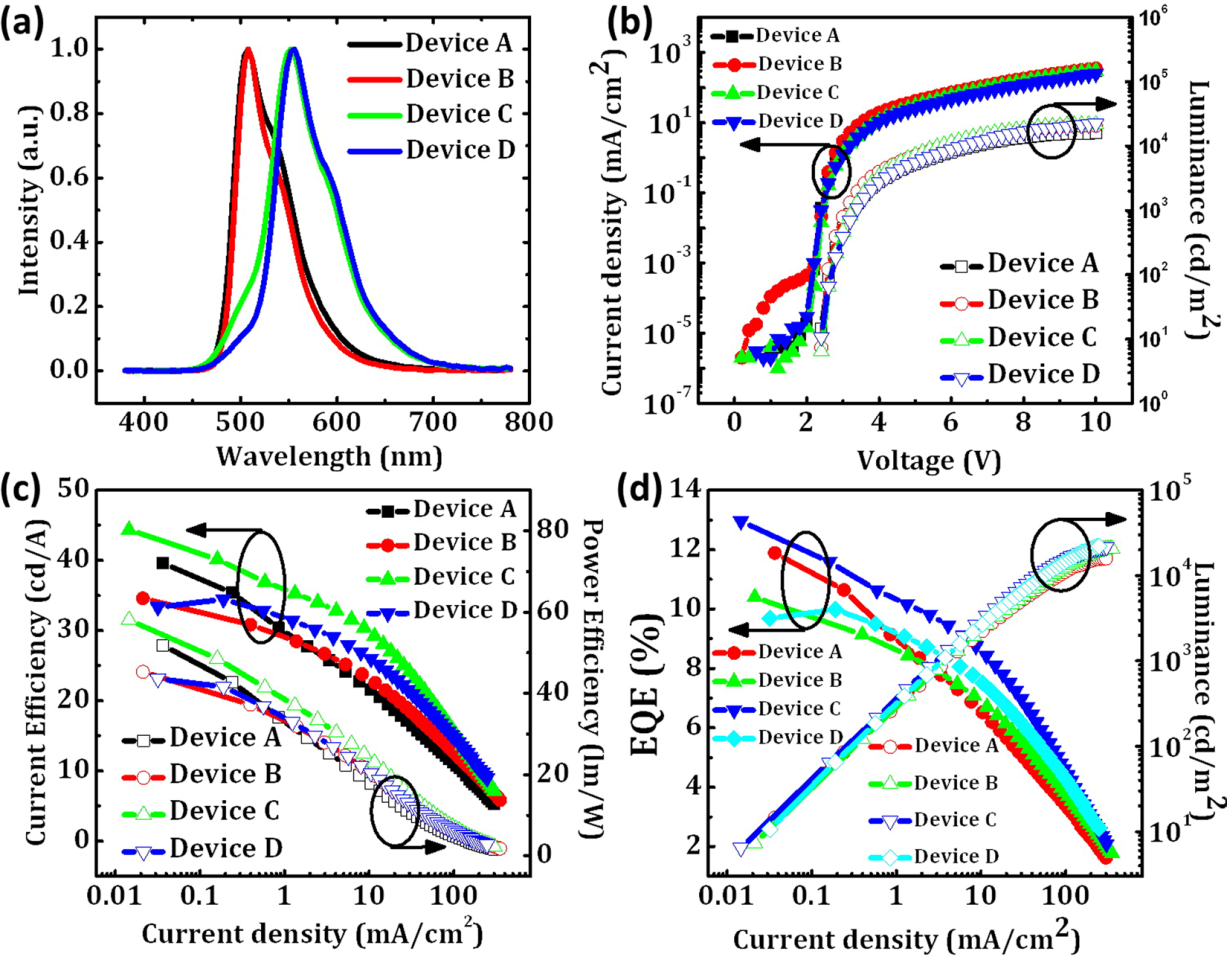
(**a**) EL spectra of the OLED devices. (**b**) current density and luminance versus voltage characteristics. (**c**) current efficiency and power efficiency versus current density characteristics. (**d**) EQE and luminance versus current density characteristics.

**Figure 4:**
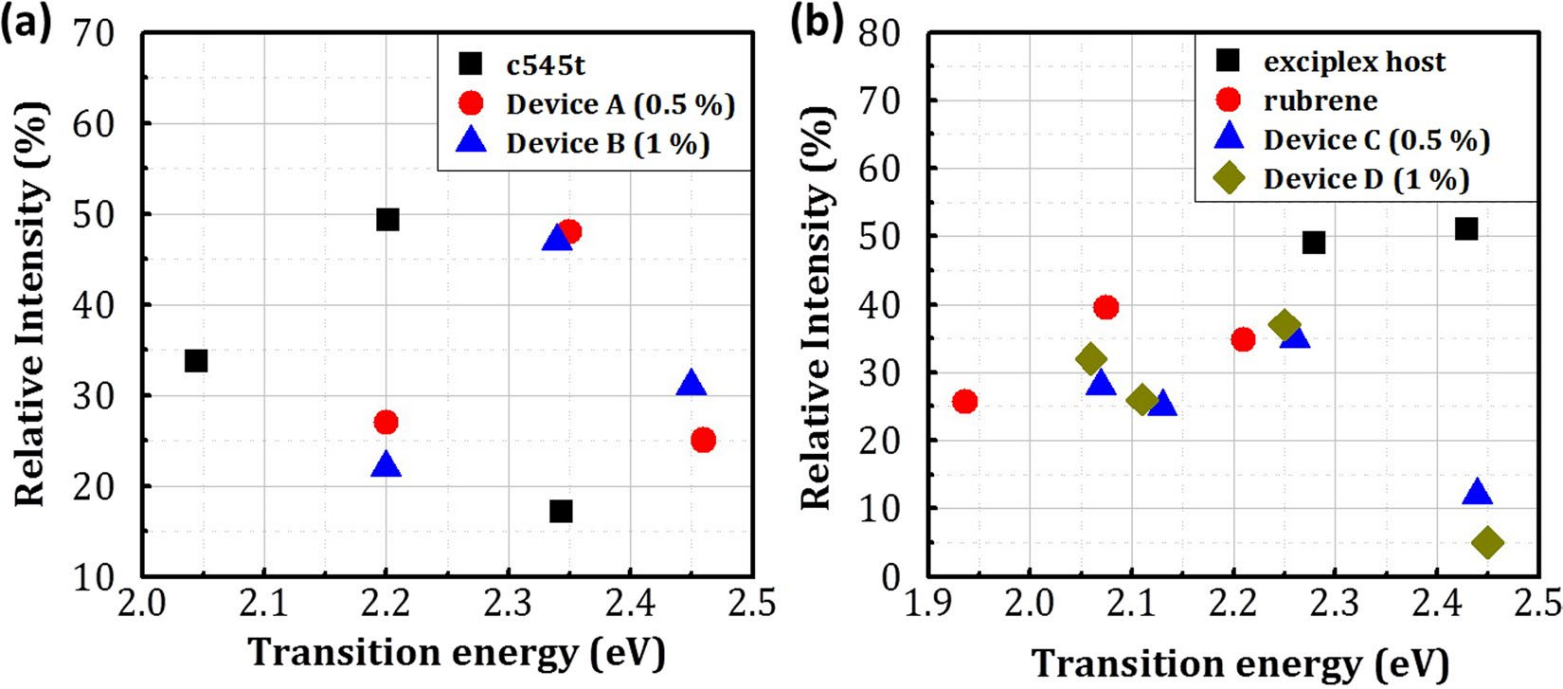
Transition energies and the corresponding relative intensities of multi-peaks fitting results using a Gaussian function. (**a**) Gaussian fitting results of PL spectrum of c545t thin film and EL spectra of c545t based OLEDs. (**b**) Gaussian fitting results of PL spectra of exciplex host and rubrene thin film and EL spectra of rubrene based OLEDs.

**Figure 5:**
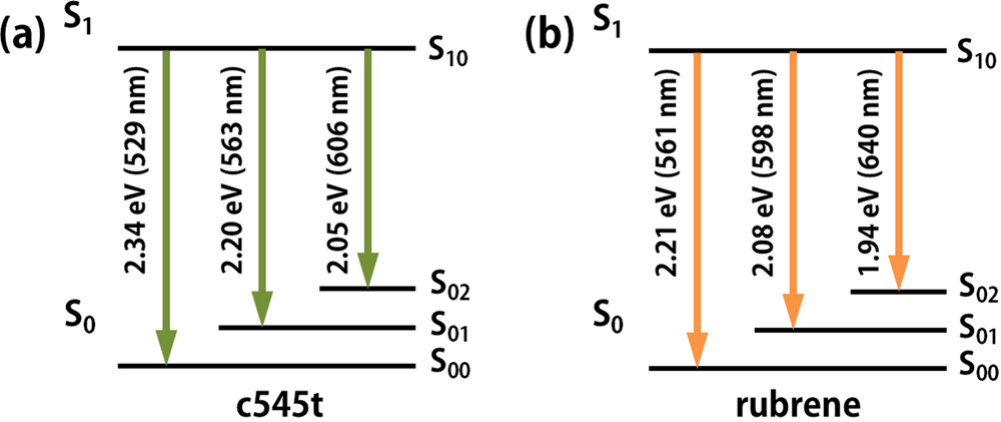
Energy level scheme of c545t and rubrene as deduced from the PL spectra.

The electroluminescence (EL) spectra of all the devices are shown in Fig. 3a. We analyzed the PL spectra (Fig. 2) and EL spectra (Fig. 3a) with a multi-peak fitting using a Gaussian function (Fig. S4) and the derived parameters are shown in Fig. 4 and Table S1. From this analysis, we have determined the PL spectra to have three transitions in c545t and rubrene^17^. The established energy levels are shown in Fig. 5. S_0_ and S_1_ indicate a series of singlet states which further subdivided into various vibrational states representing by adding a second subscript. As shown in Fig. 4a, the transitions in the c545t thin film were found to have the energies of 2.34 eV, 2.2 eV and 2.05 eV. These transitions have lower energies compared with those in the OLEDs since the distance between dopant molecules has increased so the π-π overlap is reduced and result in a blueshift of the spectra. No transition from the exciplex host is observed in both c545t based devices (Fig. S4d and e) indicating a good Förster energy transfer from the host to the dopant. The transitions in the rubrene thin film were found to have the energies of 2.21 eV, 2.08 eV and 1.94 eV (Fig. 5b). However, both rubrene based devices showed the emission from the exciplex host (~2.45 eV) as well as the three transitions from rubrene as shown in Fig. 4b. The appearance of exciplex emission is due to incomplete energy transfer from the host to the dopant. This is evident from the fact that the relative intensity of the emission from the host is reduced from 12% to 5% when the doping concentration increasing from 0.5% to 1% since the decreasing of the distance between host and dopant results in a higher rate constant of Förster energy transfer.

The current density and luminance versus voltage characteristics is shown in Fig. 3b. All the devices show very low turn-on voltage of 2.4 V. It is likely that the additional current appeared in device B is probably caused by the thermally activated leakage associated with device imperfections as well as the drift of the residual charge carrier in the intrinsic organic material when it is biased^18,19^. For green OLED devices based on c545t, Device A with the doping concentration of 0.5% shows higher external quantum efficiency (EQE), power efficiency (PE) and current efficiency (CE) when the current density is below 1 mA/cm^2^ (Fig. 3c,d). However, Device B with the doping concentration of 1% shows better performance when the current density is higher than 1 mA/cm^2^. The maximum CE of the Device A and Device B are 39.6 cd/A and 34.6 cd/A, respectively, which is significantly improved compared to OLED device employing conventional fluorescent material tris(8-hydroxyquinolinolato) aluminum (Alq_3_) as host for c545t (~12.8 cd/A)^14^. For yellow OLED devices based on rubrene, Device D with the doping concentration of 1% shows the maximum performances of 34.5 cd/A, 41.6 lm/W and 10.0%, which is much higher than OLED device using only rubrene as the EML (~0.4 lm/W) and OLED device based on rubrene doped with 4,4′-bis[N-(1-napthyl)-N-phenylamion] biphenyl (α-NPD) and Alq_3_ (~5.9 lm/W)^20,21^. The summary of performances of the OLED devices is shown in Table 2.

**Table 2:** Luminance (L), current efficiencies (CE), power efficiencies (PE) and external quantum efficiencies (EQE) of the OLED devices.

Device	Maximum				at 100 cd/m^2^			at 1000 cd/m^2^			CIE (x, y)
	L	CE	PE	EQE	CE	PE	EQE	CE	PE	EQE	
	cd/m^2^	cd/A	lm/W	%	cd/A	lm/W	%	cd/A	lm/W	%	
A	15870	39.6	51.9	11.9	35.4	42.8	10.6	25.8	25.3	7.8	(0.25, 0.62)
B	20640	34.6	45.3	10.4	30.8	37.3	9.1	26.7	28.0	7.9	(0.24, 0.64)
C	21620	44.4	58.1	13.0	40.2	48.5	11.6	34.0	33.4	9.8	(0.41, 0.55)
D	22170	34.5	41.6	10.0	34.5	41.6	10.0	29.1	26.9	8.4	(0.44, 0.54)

In conclusion, we have developed highly efficient green and yellow OLED device based on TADF host with a small ΔE_ST_ of 27 meV. By using the TADF host and conventional fluorescent emitter c545t and rubrene as the emissive layer, greater efficient energy transfers between the exciplex and the dopants improves device performance significantly. The high performances are result from the efficient utilization of triplet excitons generated in the TADF host through Forster transfer excite the dopants. The OLED devices show excellent performances, which are greatly improved compared to conventional fluorescent OLED devices.

## Methods

The PL spectra were measured by exciting the sample with a 325 nm He-Cd laser. The absorption spectra were measured by HP 8453 UV-visible spectrophotometer. The time-resolved PL were obtained using an oscilloscope (Agilent Infiniium) and samples were excited with a pulsed 355 nm Nd:YAG laser. Different ambient temperatures of the sample were realized using EDWARDS Cryodrive 3.0 connected with Intelligent Temperature Controller.

OLEDs were fabricated by vacuum thermal evaporation under the base pressure ~4 × 10^−6^ Torr. The active area of the OLEDs is 10.89 mm^2^. All the materials were used as purchased. The luminance, EL spectrum and current density versus voltage (J-V) characteristics of the OLED device were measured using Spectrascan 650 spectrometer connected with Keithley 236 source measure unit under room temperature. The EQE was calculated from spectral luminance intensity in the forward angle direction of the OLED device with a Lambertian-pattern assumption.

### Data availability

The datasets generated and/or analyzed during the current study are available from the corresponding author on reasonable request.

## Electronic supplementary material

Supplementary Information
