# Supplementary material for: Thermally Activated Delayed Fluorescence Host for High Performance Organic Light-Emitting Diodes
Source: Sci Rep. 2018 Jun 11;8:8832. doi: 10.1038/s41598-018-27238-y (PMC5995865; doi:10.1038/s41598-018-27238-y)
Supplement: Supplementary file 1 — Supplementary Information [file 41598_2018_27238_MOESM1_ESM.pdf]

Supplementary Information

Thermally Activated Delayed Fluorescence Host for High Performance Organic Light-Emitting Diodes

Lu Zhang and Kok Wai Cheah*

Department of Physics and Institute of Advanced Materials, Hong Kong Baptist University, Kowloon Tong, Hong Kong SAR, China

ABSTRACT

Thermally activated delayed florescence (TADF) materials can be an efficient host in organic LED (OLED). It is because it is possible to couple energetically the emission energy level of a dopant to the energy levels in the TADF material. In this work fluorescent emitters 2,3,6,7-tetrahydro-1,1,7,7,-tetramethyl-1H,5H,11H-10-(2-benzothiazolyl)quinolizino-9,9a,1gh coumarin (c545t) and 5,6,11,12-tetraphenyltetracene (rubrene) were used as dopants in a blended TADF host; tris(4-carbazoyl-9-ylphenyl)amine (TCTA) with 2,4,6-tris(3'-(pyridin-3-yl)biphenyl-3-yl)-1,3,5-triazine (Tm3PyBPZ). The blended TADF host has an energy difference between the singlet and triplet excited states (ΔE_{ST}) around 27meV with the yield of reverse intersystem crossing (Φ_{RISC}) nearly 100 %. This high Φ_{RISC} yield enhances the OLED performance with the c545t doped OLED having 11.9% external quantum efficiency and 10% for the rubrene doped OLED.

*E-mail: kwcheah@hkbu.edu.hk

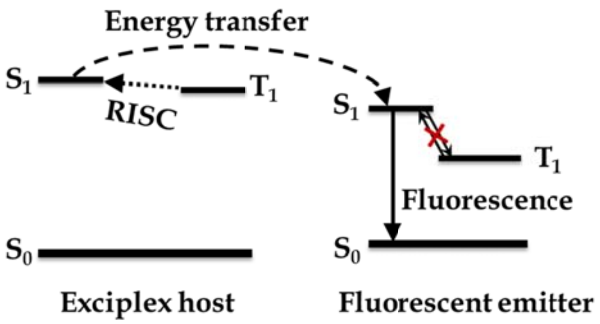

Figure S1 Schematic of exciplex functioning as host for fluorescent dopant.

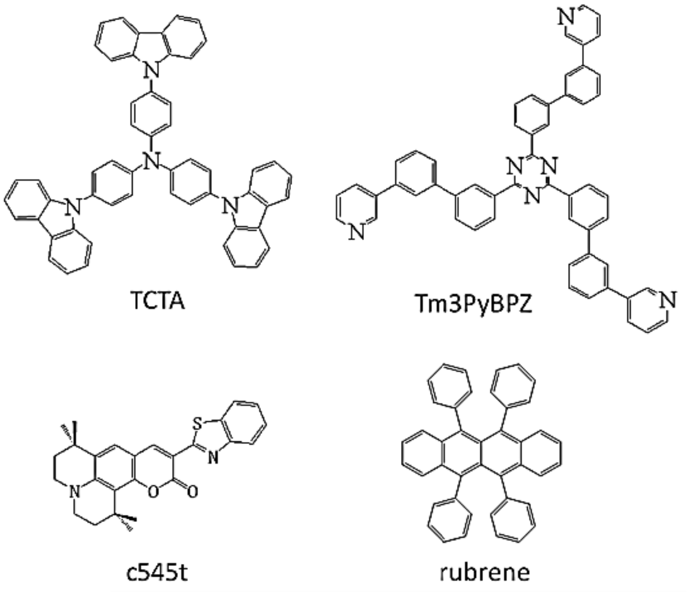

Figure S2. Chemical structures of TCTA, Tm3PyBPZ, c545t and rubrene.

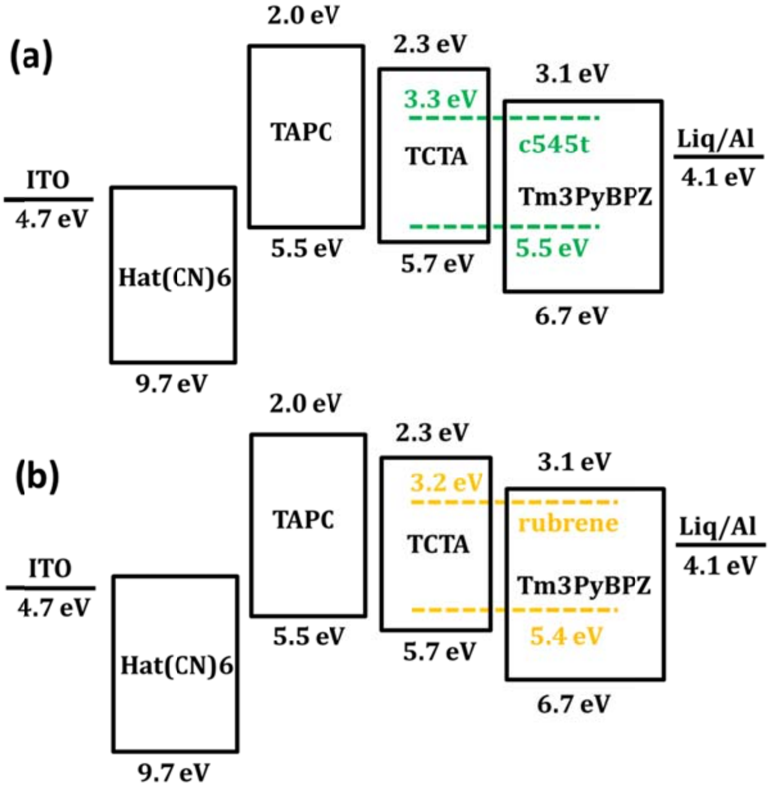

Figure S3. Energy level diagrams of OLED devices: (a) c454t based OLEDs. (b) rubrene based OLEDs.

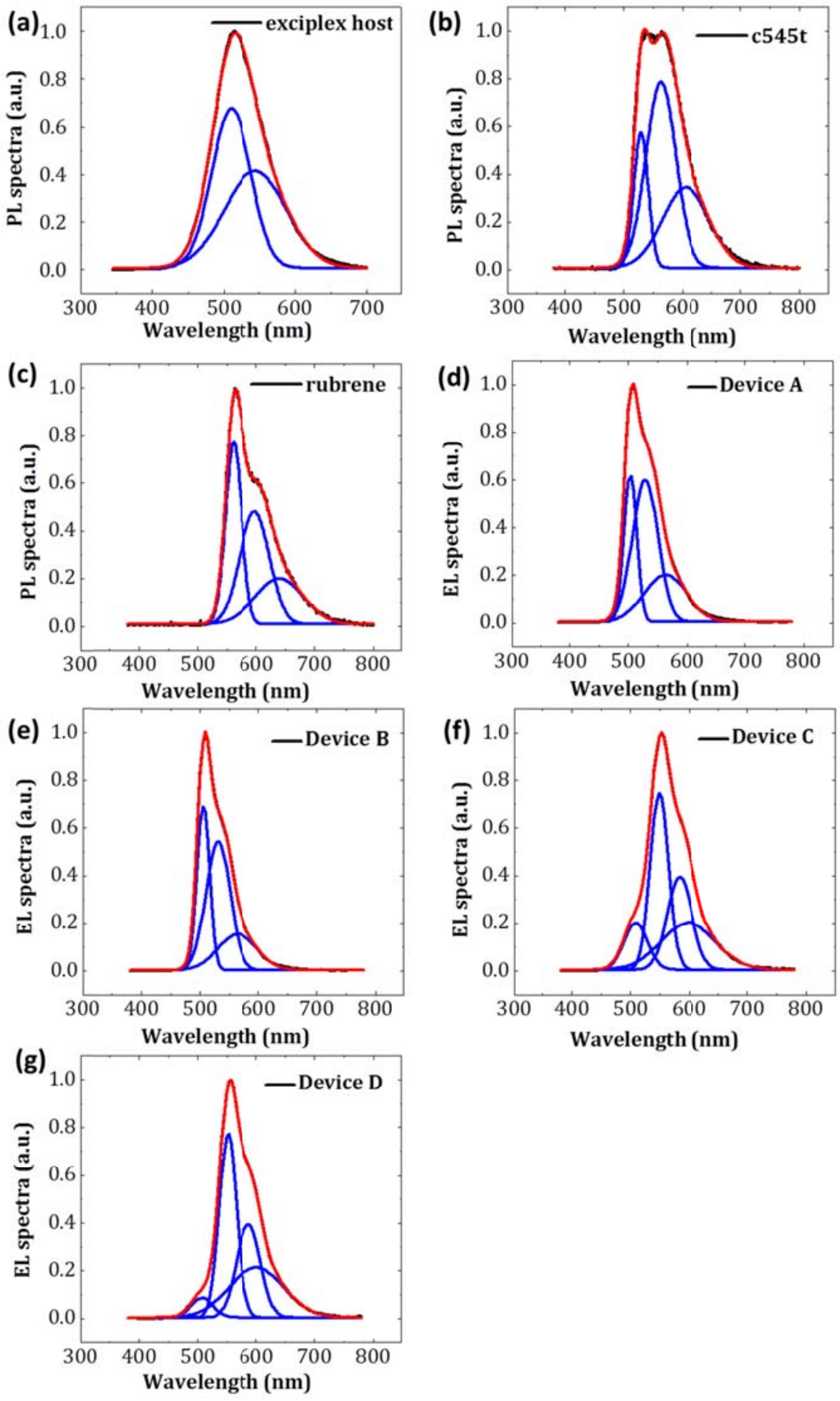

Figure S4. Multi-peaks fits with a Gaussian function. The black lines and red lines are experimental results and fitting results, respectively. The blue lines are Gaussian distributions. (a)-(c) Multi-peaks fitting of PL spectra in solid state: (a) exciplex host (50 nm), (b) c545t (50 nm), and (c) rubrene (50 nm). (d)-(g) Multi-peaks fitting of EL spectra of OLED devices at 2.6 V: (c) Device A, (d) Device B, (e) Device C, and (f) Device D.

Table S1. Wavelengths, transition energies and relative intensities of multi-peaks fitting results using a Gaussian function.

Structure		Wavelength (nm)	Transition energy (eV)	Relative intensity (%)
PL spectra	exciplex host	510	2.43	51
		544	2.28	49
	c545t	529	2.34	17
		563	2.20	49
		606	2.05	34
	rubrene	561	2.21	35
		598	2.08	40
		640	1.94	26
EL spectra	Device A	503	2.46	25
		528	2.35	48
		563	2.20	27
	Device B	505	2.45	31
		530	2.34	47
		564	2.20	22
	Device C	509	2.44	12
		549	2.26	35
		583	2.13	25
		599	2.07	28
	Device D	507	2.45	5
		551	2.25	37
		587	2.11	26
		601	2.06	32
